# It’s a Man’s World? Gender Spillover Effects on Performance in a Male-Dominated Industry

**DOI:** 10.3389/fsoc.2021.677078

**Published:** 2021-08-05

**Authors:** Charlotte Kräft

**Affiliations:** Management Department, University of Paderborn, Paderborn, Germany

**Keywords:** gender spillover, appraisal data, male-dominated industry, individual performance, fixed-effect

## Abstract

Getting more women into male-dominated industries has become the nucleus of public debate in many industrialized countries. However, it is still not clear how growing female representation impacts the individual performance of workers in these sectors. The research setting of this study is the Norwegian oil industry as a typically male-dominated sector. Using a fixed-effects regression model, the present paper investigates two different constellations: 1) how growing female representation impacts the individual performance of workers at the same hierarchical level (within-ranks); 2) how growing female representation at the next highest rank impacts the performance of subordinated workers (downward-flowing). Consistent with prevailing theory, the within-ranks analysis reveals that the performance of men in relation to a higher share of female peers follows a cubic pattern. This shows that men’s performance is the highest in gender-balanced teams. For women, this relationship cannot be confirmed. In terms of downward-flowing effects, female supervisors in this particular industry are estimated to have a negative effect on the performance of both, men and women. This result on negative downward-flowing effects requires a deeper analysis on the corporate cultural background.

## Introduction

In most industrialized countries, there is an unambiguous political consensus that women should be a vital part of the labor market (Verick, 2018). Among others, numerous political initiatives have been launched to get more women into traditionally male-dominated fields, such as Science, Technology, Engineering, and Math (STEM)-related professions (Levere, 2018). Stylized facts indicate that there is a substantial backlog for women – in OECD countries, only 24% of the female students graduate from university in a STEM-subject (OECD, 2018a). Furthermore, among graduates with science degrees, 71% of men but only 43% of women work as professionals in the physics, mathematics, and engineering spheres (OECD, 2018b). Hence, the typically male-dominated industries have become the nucleus of public debate regarding initiatives for greater women’s labor market participation.

Consequently, company leaders in those industries are increasingly more frequently confronted with requirements on gender-equal hiring and employment practices. From a theoretical point of view, gender diversity is, under specific conditions, supposed to enhance performance (Lazear, 1999a). However, especially for companies confronted with the call for gender equality, it is important to understand how increasing gender diversity impacts the individual performance of employees and whether the theoretical prediction of a positive relationship really occurs in practice. The bottom line is that they need to know whether the marginal benefits of gender diversity outweigh the related marginal costs.

Surprisingly, only sparse literature is available that analyzes gender in the context of a male-dominated industry. Certainly, there is a broad strand of the extant literature about women on management boards or, more generally, in leadership positions, which (still) represent a certain type of a male-dominated field (e.g., Bertrand and Hallock, 2001; Albanesi et al., 2015). However, when it comes to male-dominated industries, only a limited number of gender-related studies exists. While most existing studies analyze the gender pay gap and pay-related factors, little is known about the impact of growing female representation on certain outcome variables (which is known as the “*gender spillover effect*”). Indeed, some questions naturally arise in this context: e.g., Do women actually make a difference in a male-dominated field? And, simply put, is having more women always for the better? Or is there a particular threshold – some sort of “diversity saturation” – at which having more women in an industry does not bring about the desired performance gains? In this paper, I aim to answer these questions.

To accomplish this aim, I analyze possible gender spillover effects in a male-dominated industry. In this context, the spillover effect can loosely be defined as the impact of growing female representation on an outcome variable – e.g., on individual performance. Gender spillover effects can further be distinguished into two different types: 1) *downward-flowing effects* (from higher-ranking to lower-ranking individuals) and 2) *within-ranks effects* (flows within the same hierarchical rank).

For within-ranks effects, I build on Lazear’s (1999a) considerations about diversity-performance relationships in global teams. Generally, Lazear’s theory indicates performance gains through growing female representation in a male-dominated sector. Yet, there are adaptation processes involved that cause transitions costs (Lazear, 1999b). In contrast, the value threat approach describes the situation in which females, who belong to a numeric minority in work groups of high status, feel threatened by other women and as a result, have a competitive attitude towards them (Duguid, 2011; Duguid et al., 2012). Thus, the value threat approach implies negative downward-flowing spillover effects for female workers. Former studies on gender spillover effects have investigated linear relationships, exclusively. However, this study finds that within-ranks effects for men go beyond linearity and can be described as cubic. As a result, it adds new insights to the existing research on gender spillover effects.

The fact that my work analyzes the *individual performance* outcome variable is truly exceptional for several reasons. Information on individual performance is scarcely available because this is sensitive information that most companies and individuals are reluctant to share. Simultaneously, the significance of (individual) performance data is very high. Usually, in a private sector setting, nearly all employment data – such as promotions, dismissals, or wages – are primarily driven by (individual) performance. One of the main criticisms concerning performance appraisal data is that they might contain a bias against women. Existing research yields mixed results. For example, Bartol (1999) examines several field and experimental studies on gender bias in performance ratings and the findings are mixed. On the other hand, Ibarra et al. (2010) and Blau et al. (2013) confirm that there is a bias against women in performance appraisals. While I cannot rule out the possibility of biased performance appraisals for women in my data, I refer to Blau and DeVaro’s (2007) justification – if there is a bias against women in performance ratings, then this serves to decrease the “unexplained” gender gap in performance, which, in turn, yields a conservative estimate of this difference. Following this notion, if a bias against women does exist in the performance appraisals that constitute the data for this study, then the coefficients in my estimations would be underestimated. The effect would be more positive for men and more negative for women. Bearing that in mind, my findings must be treated with caution.

## Literature Review on Gender Spillover Effects

### Literature on Gender Diversity in Teams and Performance

There is a broad strand of literature on the link between gender diversity and team performance. Since my study scrutinizes gender spillover effects on *individual* performance, I will discuss this literature strand only briefly. Overall, there is only limited meta-analytic evidence of a positive link between gender diversity and team performance (Kochan et al., 2003; Pitts and Wise, 2010). Many studies reveal that there is no statistically significant relationship between gender diversity and team performance (Bowers et al., 2000; Webber and Donahue, 2001; Horwitz and Horwitz, 2007), while some studies find a significant negative effect of gender diversity on team performance (Milliken and Martins, 1996; Williams, 1998; Mannix and Neale, 2005; Bell et al., 2011). When it comes to the study design, a part of the prior research is based on field data (e.g., Pelled, 1996; Pelled et al., 1999; Lee and Farh, 2004; Chowdhury, 2005; Wegge et al., 2008; Herring, 2009) Only few of these studies convincingly address the issue of reverse causality. To overcome endogeneity problems, the effect of gender diversity on team performance has also been studied in laboratory experiments (e.g., Dufwenberg and Muren, 2006; Pearsall et al., 2008; Ivanova-Stenzel and Kuebler, 2011). In a field experiment conducted by Hoogendoorn et al. (2013), the authors manipulated the gender composition of undergraduate student teams. These student teams were tasked to start up a venture as part of their curriculum. Contrary to the findings discussed so far, the authors find that teams with an equal gender mix yield a higher performance (in terms of sales and profits) than male-dominated teams. To sum up, findings on the link between gender diversity and team performance are mixed.

To the best of my knowledge, there is only one study that explores spillover effects on individual performance – Pazy and Oron (2001) – which analyzes the relationship between gender composition and performance evaluation in the Israeli Defense Forces for the year 1995. Specifically, they use individual performance appraisal data for 3,014 high-ranking officers (2,500 male and 514 female officers). Consequently, the Israeli officer arena is considered to be a male-dominated working environment. Pazy and Oron (2001) find that female performance is rated lower when less women are in the same unit. On the contrary, when the share of females exceeds the share of men within the same unit, female performance is rated higher than male performance. The performance of men, however, does not change as a result of their proportion in a unit. Theoretically, Pazy and Oron (2001) base their study on Kanter’s (1977) sociological concept of tokenism, interpreting their findings in line with the following theoretical assumption – growing representation improves the status of a minority.

### Literature on Downward-Flowing Spillover Effects

When it comes to downward-flowing gender spillover effects, there is – to the best of my knowledge – no study that addresses the link between female leadership and individual performance in a male-dominated sector. Yet, there are studies that scrutinize downward-gender spillover effects on certain employment-related outcome variables. These outcome variables can help structure the previous literature along a typical career progression path. First, there are studies that analyze spillover effects on *hiring* (Bagues and Esteve-Volart, 2010; Bednar and Gicheva, 2014), which typically represents the first step in an individual’s professional career. Second, some studies scrutinize spillover effects on *promotion*, characterizing the next career step after an individual has been hired (Bell, 2005; Farrell and Hersch, 2005; Blau and DeVaro, 2007; Elsaid and Ursel, 2011; Matsa and Miller, 2011; Kurtulus and Tomaskovic-Devey, 2012; Karaca-Mandic et al., 2013; Kunze and Miller, 2017). Third, a wage increase is usually linked to promotion. Hence, another strand of research analyzes spillover effects on *wage levels* (Blau and DeVaro, 2007; Cohen and Huffman, 2007; Cardoso and Winter-Ebmer, 2010; Flabbi et al., 2019). Finally, there are studies that focus on the link between female leadership and distinctive *employment outcomes*, such as the share of part-time work (Devicienti et al., 2019) or the implementation of female friendly policies (Gagliarducci and Paserman, 2015).

In the following, only the direction of the spillover effect – positive, negative or insignificant – which is found by the aforementioned studies will be explained. To start with, both studies about spillover effects on hiring yield heterogeneous results – while Bagues and Esteve-Volart (2010) find negative spillover effects on hiring for both females and males, Bednar and Gicheva (2014) find insignificant effects. When it comes to promotion, all studies suggest that there are predominantly positive downward-flowing spillover effects for females (Bell, 2005; Matsa and Miller, 2011; Kurtulus and Tomaskovic-Devey, 2012; Karaca-Mandic et al., 2013; Kunze and Miller, 2017). However, the existence of gender diversity quota rules, which can “crowd-out” these positive downward-flowing effects, might be conflicting, especially when it comes to the promotion to executive ranks (Farrell and Hersch, 2005). Studies on wage confirm that there are positive downward-flowing spillover effects for women, namely female leaders reduce gender-based wage differentials (Cohen and Huffman, 2007; Cardoso and Winter-Ebmer, 2010). However, this happens through different mechanisms; while Cohen and Huffman (2007) find that men’s wages are reduced by the existence of female leaders, Cardoso and Winter-Ebmer (2010) provide evidence that women’s wages increase once a company’s management team changes from being led by a man to being led by a woman. Devicienti et al. (2019) find positive downward-flowing effects, in the sense that female leaders are significantly more likely to limit the employment of involuntary part-time workers and grant part-time arrangements to employees who request them. In the same vein, Gagliarducci and Paserman (2015) reveal that female leaders are more likely to implement female-friendly policies. Thus, they also find positive downward-flowing effects, at least for women.

In sum, the results of previous studies on spillover effects differ and depend on the outcome variable that is analyzed. The bottom line is that there is no study that analyzes both spillover effects types on individual performance in a male-dominated setting. While my research endeavor has the biggest overlaps with Pazy and Oron (2001), my project stands out through some of its notable features. First, I extend my existing work not only on within-ranks effects but on downward-flowing spillover effects for females and males. Second, my methodological approach does not rely on variance analyses; instead, I estimate a range of fixed-effects regression models. Furthermore, I analyze data from the more recent years, 2008 to 2014, thereby adding a substantial contribution to the existing research. Against a background in which many initiatives for fostering women in leadership and for promoting (gender) diversification have been introduced in recent years, an analysis of data that originate from a more current timeframe might be particularly instructive. Finally, it should be noted that individual performance data are rarely available. At the same time, individual performance is a variable that impacts all steps of a professional career, and is thus a highly relevant variable for company performance. Consequently, I make a valuable contribution to the sparse academic discussion about spillover effects on performance.

## Theoretical Background and Hypotheses

### Within-Ranks Spillover Effects: Gender Diversity in Teams and Performance

Lazear (1999a) provides certain theoretical predictions about diversity-performance relationships in teams. Originally, his theory focuses on teams that have members from diverse cultures. Nevertheless, this can easily be transferred into the context of this study because men belong to a distinct (business) culture – i.e., to a male-dominated culture that shares a set of certain practices, beliefs, and communication and decision rules, which differentiate it from the distinct (business) culture of women (Neculaesei, 2015). Lazear’s (1999a) theory follows a basic principle that stipulates that a complementariness must exist between workers of diverse cultures, which is crucial enough to overcome the costs of bringing them together. Lazear (1999a) further argues that there are three factors that are decisive in forming a diverse team: 1) various groups have information or skill sets that are disjoint; 2) this information or skill is relevant to the other group(s); and 3) they are communicated adequately so that these can be learned by the other group(s) at a reasonable cost. When these preconditions are met, (gender) diverse teams result in performance gains.

I assume that in the business context of this study these preconditions are fulfilled. For instance, an efficient selection of personnel guarantees that workers of both genders who have complementary skill sets are placed into one team and that no overlaps occur. In this regard, some studies reveal women’s disposition for specific social and emotional skill sets (Groves, 2005). These competencies might facilitate team work and team output together with more “agentic” behavior that is found to be pronounced for men (Eagly et al., 2000). Furthermore, the communication between these members is realized through regular cooperation in a daily working routine so that knowledge transfer is guaranteed. Since the business setting of this study is characterized as an environment that is rather gender imbalanced, I assume that a minority and majority culture adaptation process takes place. The prevailing theory suggests that workers who belong to a minority group tend to adopt the majority culture when the minority group accounts for only a small proportion of the entire workforce. However, it is unlikely that the majority group would adopt aspects of the minority culture. Moreover, the introduction of minority cultures causes transition costs that result from an expected transition period (Lazear, 1999b).

Transferring these principles to the business setting of this study, the performance gains obtained through gender-diverse teams might be characterized by transition periods. These adaptation processes can be understood as trading processes between both groups. In addition, efficient intra-group processes depend on the thresholds that mark the minority and majority proportions of the workforce. Bearing these dynamics in mind, the performance gains obtained through gender-diverse teams would most likely not be linear. I hypothesize that the individual performance of workers of both genders will be positive in gender-balanced teams. However, initial trading and adaptation processes might slow down or even decrease the performance surge. Beyond a certain threshold, when workers of one gender dominate the team, individual performance will decrease again. This relationship applies to the individual performance of both, men and women. Consequently, I formulate the first hypothesis:

**Hypothesis 1:** *The relationship between the individual performance of men and women and the growing share of female peers will be non-linear in a male-dominated industry. Individual performance will follow a cubic pattern as the share of female peers grows.*

### Downward-Flowing Spillover Effects: Female Leadership in a Male-Dominated Setting

Based on Phelp’s (1972) statistical discrimination model, Flabbi et al. (2019) developed a simple signal extraction theory in which disparities are created by supervisors’ insufficient information about workers’ productivity and where the supervisors’ gender plays a crucial role. More precisely, Flabbi et al. (2019) postulate that supervisors are more capable of assessing workers’ skills of the same gender. This might be due to better communication and better ability at personal relationships, or a common cultural background shared by individuals of the same gender. Applying this theory to downward-flowing spillover effects for men, female supervisors in general might be more accurate in interpreting signals of productivity from female workers due to the same cultural background they share and related communication advantages. Thus, I hypothesize that the lack of interpreting productivity signals of male workers has a discouraging effect on their performance, resulting in a performance decline as the share of female supervisors grows. I further assume that the link between female supervisors and men’s performance is linear. Consequently, the second hypothesis can be formulated as follows:

**Hypothesis 2**: *There will be a negative relationship between men’s individual performance and a growing share of female supervisors in a male-dominated industry. This relationship is linear.*

Transferring the principles of the signal extraction theory by Flabbi et al. (2019) to the relationship between female supervisors and female workers would certainly result in performance gains for female workers. However, Staines et al. (1974) introduced the so-called queen bee phenomenon to describe a situation that women who are individually successful in male-dominated industries and accomplish executive positions are more likely to endorse gender stereotypes. That is, they tend to consider the women they supervise as competitors with negative attitudes towards them (Derks et al., 2011a). In a similar vein and more accurately depicting the background of my study, the value threat approach implies that the relationship among women in male-dominated sectors can be described as being rather competitive than supportive (Duguid et al., 2012). Precisely, the value threat approach suggests that women belonging to a numeric minority might feel threatened or “endangered” by other women if the other women either have better qualifications (competitive threat) or endorse negative stereotypes about female management qualifications (collective threat) (Duguid et al., 2012). Ultimately, this behavioral pattern can be ascribed to the individual fear of women of not being appreciated within a high-status work group. As a result, downward-flowing spillover effects on women are assumed to be negative. Contrasting to the model of Flabbi et al. (2019), female supervisors are unwilling to interpret women’s productivity signals in a male-dominated sector. I hypothesize that the value threat approach is salient in the industry setting of my study since it is a traditionally male-dominated field where masculine communication and decision-making patterns might dominate as well as related power and network structures might be salient. These should even be more pronounced for the executive ranks and imply a distinct value threat for women. Assuming that female leadership in a male-dominated industry is tied to the value threat approach, the female leadership style has a discouraging, demotivating effect on the women below which in turn will result in diminishing female individual performance.

Hence, my third hypothesis reads as follows:

**Hypothesis 3**: *There will be a negative relationship between women’s individual performance and a growing share of female supervisors in a male-dominated industry. This relationship is linear.*

## Data Set and Descriptive Statistics

### Data

For this study, I analyzed a comprehensive personnel panel data set from the Norwegian crude oil and gas industry for the 2008–2014 period. The data set was compiled by an international consulting firm that specializes in remuneration. It included personnel data from six Norwegian companies, whose major business activity was the production of crude oil and gas. These companies did not necessarily need to be of Norwegian origin – their headquarters could have been located in another country. A total of 6,871 individual workers at the full-time or equivalent level and 19,164 observations were associated with these companies. Only 24.7% of the observations were obtained from female workers, while the majority, 75.3%, of the observations were obtained from male workers. Underlining the male-dominance in this sector is the fact that this number of men is greater than the national average for Norway’s entire private sector, where men comprise 63.4% (National Statistical Institute of Norway, 2017).

The fact that my data are Norwegian is particularly interesting because Norway is known as a pioneer with regards to gender equality policy (Milne, 2018). Norway was the first European country to launch a quota law for female representation on company boards in 2003. Hence, it is reasonable to expect that Norway would have a long tradition in female labor market participation. Nevertheless, the “Nordic gender-equality paradox” still proves the phenomenon that, especially in very advanced Nordic countries, the distribution of women in superior job positions (particularly in male-dominated fields) is still rather uneven (Sanandaji, 2016). Occasionally, the Nordic gender-equality paradox is attributed to the generous welfare policy in the Nordic countries, which makes it possible for women to be on parental leave or to occupy lower-ranked positions with lower wages (Sanandaji, 2016). Against this background, studying Norwegian data could provide meaningful insights.

The data set was used to allocate the employees according to nine different functional areas, including geosciences or project engineering. Individuals were further distinguished based on their job location (offshore, onshore production, and onshore non-production). Besides gender, the data set included other human capital variables, such as age and tenure.

The major advantage of the data set lies in the fact that it made detailed information on remuneration (e.g., fixed wage, actual and planned bonuses) and hierarchical rank available. The hierarchical rank of each individual represented the responsibilities associated with their position and related to a certain remuneration level or fringe benefit amount. Employees who held a managerial responsibility over a business unit belonged to the first rank in the hierarchy. The employees in simple technical jobs that lacked managerial responsibility were ranked lowest (seventh rank).^1^ Thus, the hierarchical structure did not include the highest and lowest ranks of wage pyramids, which are typically held by Chief Executive Officers (CEOs) and unskilled workers, respectively. Based on individual performance, supervisors determined their employees’ actual bonus wages. Therefore, the actual bonuses reflected supervisors’ evaluations of employee’s performances during the previous business. The performance evaluation is based on three principles: how well the tasks are executed, the individual’s output compared to what is considered normal in the job, and how accurately the worker follows instructions and regulations. The performance evaluation is relative in nature.

### Descriptive Statistics and Variables

Table 1 presents the descriptive statistics of the main variables for men and women, separately.

**TABLE 1:** Descriptive statistics of the main variables.

Variable	Definition	Men in the sample			Women in the sample		
		Mean (sd)	Min	Max	Mean (sd)	Min	Max
Individual performance	Degree of individual target achievement	1.074 (0.250)	0	2.637	1.057 (0.229)	0	2.179
Female peer share	Share of female team members within the same rank	0.233 (0.079)	0	0.667	0.270 (0.088)	0.050	1
Female boss share^a^	Share of female supervisors in the next higher rank	0.196 (0.086)	0	1	0.217 (0.087)	0	1
Age	Age of the individual worker	45.332 (9.982)	22	70	39.790 (8.550)	22	67
Job function	1 = commercial, 2 = discipline engin., 3 = general engin., 4 = geosciences, 5 = HSE, 6 = production engin., 7 = project engin., 8 = petroleum engin., 9 = well engin.	—	1	9	—	1	9
Job location	1 = offshore, 2 = onshore non-production, 3 = onshore production	—	1	3	—	1	3
Rank	Hierarchical rank, 1 = highest rank, 7 = lowest rank	4.134 (1.359)	1	7	4.580 (1.345)	1	7
Company	Employing company	—	1	6		1	6
Year	Business year	—	2008	2014		2008	2014

*Note.* Descriptive statistics were obtained from 19,164 observations; 14,429 men and 4,735 women.

a Descriptive statistics were obtained from 17,983 observations, 13,428 men and 4,555 women (The number of observations was reduced to 17,983 because workers at the highest hierarchical rank - rank 1 - do not have any supervisors.

The dependent variable of my analysis was individual performance. In the following analysis, I defined individual performance as actual bonus amount divided by the target bonus amount. Thus, individual performance signified the degree of individual target achievement. I interpreted performance as purely actively-driven by the respective individual worker. Its mean value for men (1.07) implied that most male workers have over-accomplished their target by 7 percentage points, whereas women have over-accomplished their target by 6 percentage points. Yet, both minimum values for men and women displayed that some individuals’ performance is zero while the maximum value of 2.64 (men) and 2.18 (women) showed that there are also “high performers” who have more than doubled their target performance. However, the standard deviations (0.25 and 0.23) implied that most performance values are rather close to the mean.

Next to individual performance, two additional essential variables were generated. The female peer share variable denoted the share of female peers within a hierarchical rank (per year and per company) and the female boss share variable indicated the share of female supervisors in the next higher hierarchical rank (per year and per company). Any variations of the female peer share and female boss share variables were indicated by different years, companies, and hierarchical ranks. Since I did not have any information about the share of females in specific departments or business units, I needed to construct both variables on the basis of the entire hierarchical rank. Hence, for the female boss share variable, I was unable to identify the direct supervisors of workers (similar to Blau and DeVaro, 2007; Karaca-Mandic et al., 2013; Kunze and Miller, 2017). The female boss share variable refers to the female representation at the next hierarchy level and is rank-specific. By using this noisy measure, the effects of female leadership deep within organizational hierarchies can be quantified (Kunze and Miller, 2017). The summary statistics of female peer share showed that for men, on average, 23.3% of the peers within the same hierarchical rank were female. For women, 27.0% of the peers within the same hierarchical rank were female. The average female boss share amounted to 19.6% for men, and 21.7% for women - indicating that the vast majority of supervisors were male.

The rank variable signified the hierarchical rank of an individual worker. While on average, both men and women held a position at rank 4, men’s mean rank was higher than women’s mean rank (4.13 vs. 4.58). The rank variable was used to construct the key explanatory variables: female peer share and female boss share. Furthermore, rank was also used as a control variable to account for the possible effects on individual performance that stem from different hierarchies in which individuals work. For instance, the individual performance of workers who were responsible for a team might also be linked to the ambitions of their managed team, while this was certainly not the case for employees who held simple commercial positions.

Next, the average male worker in the data set was 45.3 years old, the average female worker was 39.8 years old indicating that female workers in the data set were younger than male workers. Age was included in my analysis to account for the effects on performance that arise from a certain seniority and experience level.

Further control variables were job function and job location, displaying the field that an individual can be allocated to within a respective company. Including job function as control variable accounted for different working conditions within a functional area and for the related effects on individual performance. For instance, in some functions, individual performance can be measured more directly than in other functions. Job location was included as a proxy variable for different cooperation and communication conditions that might impact individual performance.

The company variable signified the employing company. Including the employing company as a control variable accounted for the possible effects of corporate culture and structure on individual performance.

The year variable represented the respective business year. The year was a trend variable that accounted for economic shifts and any unmeasured differences.^2^

## Empirical Analysis

### Estimation Strategy

I tested the three hypotheses using a fixed-effects model for men and women, separately. I employed the fixed-effects model because it controls for all the time-invariant differences between the individuals. As a result, the estimated coefficients cannot be biased due to the omitted time-invariant characteristics. Moreover, fixed-effects models are default methods for causal inference with longitudinal data. Besides, I applied the Hausman specification test in order to decide if a fixed-effects or a random-effects model should be used in this analysis. The Hausman test calls for the fixed-effects model.^3^

$$individual\;performance_{it}=\;\beta_{0}+\;\beta_{1}\mathrm{female peer shar}e_{\mathrm{it}}\;+\beta_{2}\mathrm{female peer shar}e_{\mathrm{it}}^{2}+\;\beta_{3}\mathrm{female peer shar}e_{\mathrm{it}}^{3}+\;\beta_{4}age_{it}+\;\;\beta_{5}age_{it}^{2}+\;\beta_{6}rank_{it}+\;\beta_{7}job\;function_{it}+\;\beta_{8}job\;location_{it}+\;\beta_{9}company_{it}+\;\beta_{10}year_{it}+\;\pi_{i}+\;\varepsilon_{it}.$$ (1)

$$individual\;performance_{it}=\;\beta_{0}+\;\beta_{1}\mathrm{female boss shar}e_{\mathrm{it}}\;+\;\beta_{2}age_{it}+\;\;\beta_{3}age_{it}^{2}+\;\beta_{4}rank_{it}+\;\beta_{5}job\;function_{it}+\;\beta_{6}job\;location_{it}+\;\beta_{7}company_{it}+\;\beta_{8}year_{it}+\pi_{i}+\;\varepsilon_{it}.$$ (2)

Regression model 1) included the share of female peers as a covariate, while regression model 2) included the share of female supervisors as a covariate. To allow for a non-linear relationship in (1), the model was expanded by adding the female peer share key variable in quadratic and cubic format. *π*_*i*_ is a company-specific fixed effect; and $\varepsilon_{it}$ is the error term. All control variables have been explained in Section *Descriptive Statistics and Variables*.

### Estimation Results

#### Within-Ranks Spillover Effects: Estimation Results

Tables 2, 3 show the results of the fixed-effects model 1) for men and women, separately. Table 2 reports the results of the fixed-effects model for men’s individual performance.

**TABLE 2:** Regression results for within-ranks effects for men.

Individual performance	(1)	(2)	(3)
Female peer share	0.268***	0.459*	−0.941**
	(0.091)	(0.249)	(0.525)
Female peer share^2^	—	−0.356	5.037***
	—	(0.412)	(1.640)
Female peer share³	—	—	−5.770***
	—	—	(1.514)
Constant	−0.483*	−0.512*	−0.409
	(2.886)	(0.289)	(0.292)
Observations	14,429	14,429	14,429
*R* ^2^	0.014	0.013	0.014

Notes. Table reports coefficients for three different estimations: columns 1) linear, 2) quadratic, and 3) cubic, with individual performance as the dependent variable. Robust standard errors are included (in parentheses). Estimations include age, age^2^, hierarchical rank, job function, job location, company, year controls, and company fixed effects.

* *p* < 0.10.

** *p* < 0.05.

*** *p* < 0.01.

**TABLE 3:** Regression results for within-ranks effects for women.

Individual performance	(1)	(2)	(3)
Female peer share	0.980	0.740***	0.591
	(0.099)	(0.265)	(0.807)
Female peer share^2^	—	−0.902***	−0.487
	—	(0.309)	(2.032)
Female peer share³	—	—	−0.326
	—	—	(1.478)
Constant	0.574	0.482	0.503
	(0.451)	(0.449)	(0.454)
Observations	4,735	4,735	4,735
*R* ^2^	0.032	0.027	0.028

Notes. Table reports coefficients for three different estimations: columns 1) linear, 2) quadratic, and 3) cubic, with individual performance as the dependent variable. Robust standard errors are included (in parentheses). Estimations include age, age^2^, hierarchical rank, job function, job location, company, year controls, and company fixed effects.

* *p* < 0.10.

** *p* < 0.05.

*** *p* < 0.01.

**FIGURE 1:**
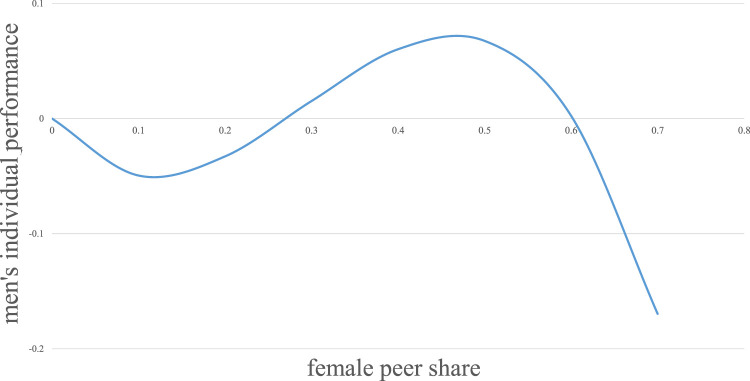
Cubic regression results for individual performance and female peer share for men.

Column 3) entails the linear, quadratic, and cubic terms, achieving significance at the 1%-level for the quadratic and cubic coefficients and significance at the 5%-level for the linear term. For ease of interpretation, Figure 1 visualizes the cubic relationship between the individual performance and the share of female peers for men. Since the maximum female peer share value for men is 0.67 (see Table 1 for descriptive statistics), the analysis is restricted to this range of female peers.

Figure 1 visualizes that, overall, there are only intermediately positive returns to the individual performance of men as the share of female peers grows. This is true when the share of female peers is between 0.27 and 0.60. Before and after this female peer share range, the individual performance of men is associated with negative returns. Specifically, men’s performance initially declines for a female peer share until approx. 0.1 Then it increases, whereas its maximum point is at a female peer share of approx. 0.5. Afterwards, as the share of female peers grows, men’s individual performance declines again.

Table 3 reports the results for the within-ranks effects for women. Only the quadratic model 2) yields significant estimations (at the 1%-level). This suggests that the individual performance of women is estimated to follow an inverted U-shape pattern as the share of female peers grows. First of all, although female peers are associated with increasing the individual performance of women, there is a turning point at around 0.41 of female peers. After this value, the female peer share is linked with a decreasing individual performance.

On the basis of my analysis, I need to reject **Hypothesis 1** (i.e., *The relationship between the individual performance of men and women and the growing share of female peers will be non-linear in a male-dominated industry. Individual performance will follow a cubic pattern as the share of female peers grows.*). For men’s individual performance, Hypothesis 1 can be approved: It follows a cubic curve with growing share of female peers. However, for the individual performance of female workers the estimates of the cubic model are insignificant. Their performance is estimated to follow a quadratic, more precisely an inverted U-shape pattern as the share of female peers grows.

#### Downward-Flowing Spillover Effects: Estimation Results

Table 4 presents the estimations of the female boss share in linear format for men’s and women’s individual performance, respectively.

**TABLE 4:** Regression results for linear downward-flowing effects for men and women.

Individual performance	Men (1)	Women (2)
Female boss share	−0.412***	−0.511***
	(0.056)	(0.086)
Constant	0.127	0.877**
	(0.270)	(0.437)
Observations	13,428	4,555
*R* ^2^	0.052	0.075

Notes*.* Table reports coefficients for linear estimations with individual performance as the dependent variable. Robust standard errors are included (in parentheses). Estimations include age, age^2^, hierarchical rank, job function, job location, company, year controls, and company fixed effects.

* *p* < 0.10.

** *p* < 0.05.

*** *p* < 0.01.

Column 1) entails the fixed-effects results for men’s individual performance. The linear model for men’s individual performance indicates that female supervisors are estimated to have a negative effect on the individual performance of men. Specifically, the rise of female supervisors by one unit is associated with a decline in the performance of men by 0.41, achieving significance at the 1%-level.

Column 2) shows the regression results for the downward-flowing spillover effects for women. The estimation indicates that the rise of female supervisors by one unit is associated with a 0.51 decrease in the performance of women, achieving significance at the 1%-level.

Thus, I can confirm **Hypothesis 2** (i.e., *There will be a negative relationship between men’s individual performance and a growing share of female supervisors in a male-dominated industry. This relationship is linear.*). The estimates suggest that men do decrease their performance as the share of female supervisors grows. Specifically, the rise of female supervisors by one unit is associated with a performance decline by 0.41.

In the same vein, I can confirm **Hypothesis 3** (i.e., *There will be a negative relationship between women’s individual performance and a growing share of female supervisors in a male-dominated industry. This relationship is linear*). My analysis suggests that female supervisors are also associated with a decrease in the performance of women. Specifically, as the share of female supervisors grows by one unit, the performance of women is associated with a decline of 0.51. The effect of a growing share of female supervisors is stronger for female individual performance than for male individual performance.

### Robustness Checks

To underpin the robustness of my findings, I conducted different robustness checks for each model. First, I applied the within-ranks effects model for a limited sample of a random group of job functions. The notion was to check whether the cubic relationship between male individual performance and a growing share of female peers is robust with regards to selected work environments (see Table 5). The result of this robustness check confirms the cubic relationship between a growing share of female peers and men’s individual performance, although the magnitudes of the coefficients and their significance level differ (the significance level of the quadratic and cubic terms changed from 1 to 5%-significance level). Yet, their signs remain constant documenting the robustness of the cubic relationship for men’s performance.

**TABLE 5:** Regression results for within-ranks effects for men for a random group of job functions (Commercial, General Engineering and Geosciences).

Individual performance	
Female peer share	−1.944**
	(0.891)
Female peer share^2^	5.696**
	(2.599)
Female peer share³	−5.039**
	(2.189)
Constant	−0.305
	(0.496)
Observations	4,938
*R* ^2^	0.011

Notes. Table reports coefficients for cubic estimation, with individual performance as the dependent variable. Robust standard errors are included (in parentheses). Estimations include age, age^2^, hierarchical rank, job function, job location, company, year controls, and company fixed effects.

* *p* < 0.10.

** *p* < 0.05.

*** *p* < 0.01.

Second, I limited the female boss share below the value 0.5 since the descriptive statistics suggest that this is a critical threshold where below the majority of observations lies. I estimated the downward-flowing spillover effects model for male and female performance with this limited covariate in order to prove that the results are not driven by some outliers (see Table 6). Again, although the magnitude of coefficients slightly differs, the signs as well as significance levels stay the same confirming the negative downward-flowing effects for both, male and female individual performance.

**TABLE 6:** Regression results for linear downward-flowing effects for men and women with female boss share <0.5.

Individual performance	Men (1)	Women (2)
Female boss share <0.5	−0.380***	−0.686***
	(0.051)	(0.135)
Constant	0.144	0.123
	(0.271)	(0.270)
Observations	13,354	4,505
*R* ^2^	0.056	0.072

Notes*.* Table reports coefficients for linear estimations with individual performance as the dependent variable. Robust standard errors are included (in parentheses). Estimations include age, age^2^, hierarchical rank, job function, job location, company, year controls, and company fixed effects.

* *p* < 0.10.

** *p* < 0.05.

*** *p* < 0.01.

Lastly, I estimated both models with alternative estimation approaches, namely with a Random-Effects (RE) and an Ordinary Least Squares (OLS) regression model (see Tables 7, 8). Again, both effects, within-ranks effects for male performance and downward-flowing effects for male and female performance have been confirmed when it comes to the sign of coefficients.

**TABLE 7:** Random-effects and ordinary least squares regression results for within-ranks effects for men.

Individual performance	RE (1)	OLS (2)
Female peer share	−0.902***	−0.852***
	(0.336)	(0.277)
Female peer share^2^	3.992***	3.850***
	(1.106)	(.952)
Female peer share³	−4.365***	−4.254***
	(1.111)	(0.980)
Constant	0.845	0.830
	(0.069)	(0.054)
Observations	14,429	14,429
*R* ^2^	0.385	0.388

Notes. Table reports coefficients for cubic estimations, with individual performance as the dependent variable. Robust standard errors are included (in parentheses). Estimations include age, age^2^, hierarchical rank, job function, job location, company, year controls, and company fixed effects.

* *p* < 0.10.

** *p* < 0.05.

*** *p* < 0.01.

**TABLE 8:** Random-effects and ordinary least squares regression results for linear downward-flowing effects for men and women.

Individual performance	Men RE (1)	Men OLS (2)	Women RE (3)	Women OLS (4)
Female boss share	−0.205***	−0.200***	−0.187***	−0.166***
	(0.006)	(0.039)	(0.058)	(0.049)
Constant	0.016***	1.015***	0.991***	0.979***
	(0.053)	(0.047)	(0.105)	(0.090)
Observations	13,428	13,428	4,555	4,555
*R* ^2^	0.445	0.445	0.457	0.457

Notes*.* Table reports coefficients for linear estimations with individual performance as the dependent variable. Robust standard errors are included (in parentheses). Estimations include age, age^2^, hierarchical rank, job function, job location, company, year controls, and company fixed effects.

* *p* < 0.10.

** *p* < 0.05.

*** *p* < 0.01.

## Discussion and Conclusion

### Discussion and Implication of Results

In this paper, I analyze two different spillover effect types on the individual performance outcome variable – within - ranks effects and downward-flowing effects for a male-dominated industry in Norway. Specifically, I extend my analysis to the question whether within-ranks gender spillover effects can be more accurately described using a cubic model. Prior research on gender spillover effects assumes that spillover effects are exclusively linear.

Even though Norway is a pioneer country when it comes to gender-equality at the workplace, my findings suggest that women who are in peer and in management positions do make a difference. I find that the performance of men in relation to a higher share of female peers follows a cubic pattern. For example, it is only within a 0.27–0.60 range of the female peers share that its growth is associated with positive returns for the individual performance of men. Furthermore, it is interesting to see that the thresholds for the share of female peers at which men seem to adjust their performance levels (0.29 from decreasing to increasing performance, 0.5 from increasing to decreasing performance) mark the proportions of female peers that can be seen as minority (until 0.29) or majority thresholds (from 0.5 onwards) in a male-dominated industry. The 0.29–0.49 range, where the performance level for men increases, can be interpreted as being gender-balanced, which is in line with Lazear’s (1999b) theoretical considerations introduced in Section *Within-Ranks Spillover Effects: Gender Diversity in Teams and Performance*. Hence, my findings partially support Lazear’s theory.

In terms of downward-flowing spillover effects, female supervisors are estimated to have a negative effect on the performance of men and women. Specifically, the rise of female supervisors by one unit is associated with a decline in the individual performance of men by 0.41, and a 0.51 decline in the individual performance of women. My results for women’s individual performance suggest that the value threat approach can be applied to this situation. That is, women who are in supervisory roles in a male-dominated setting regard female subordinates as competitors and serve more as “hurdles.” Therefore, these female subordinates seem to reduce their performance levels accordingly. When it comes to the relationship between men’s decreasing performance and a growing share of female supervisors, this can be due to female supervisors’ inability to interpret men’s productivity signals as pointed out by the model of Flabbi et al. (2019) in Section *Downward-Flowing Spillover Effects: Female Leadership in a Male-Dominated Setting*. Hence, both research hypotheses on downward-flowing spillover effects could be approved.

The results of this study offer room for various interpretations. When it comes to the cubic within-ranks effects for men’s performance, the fact that a share of female peers up to 0.29 is associated with a decreasing performance of men could be a consequence of new communication and decision patterns that “female newbies” bring with them. The “recovery phase” (0.29–0.49) could then mark an adjustment phase, whereas the decreasing performance level from 0.5 onwards could signify that these new “female communication and decision-making rules” do finally dominate interaction structures that lead to social categorization, which causes conflicts and, ultimately, negatively affects outcomes. With respect to the negative downward-flowing spillover effect, typically, in a male-dominated industry, very few female employees hold supervisory positions. Consequently, women could reduce their performance once a supervisory role is taken by a “female competitor” because this may diminish the probability that any of the other women would occupy one themselves as a result. Interestingly, in a male-dominated setting, women in supervisory roles do not serve as role models or mentors who facilitate networking which is found for other industries (Athey et al., 2000). Another intuitive interpretation of the results would be that female supervisors manage their subordinates differently than male supervisors–in the sense that they are more socially-oriented and not as focused on “hard facts” such as performance (Chapman, 1975; Matsa and Miller, 2013). However, all explanations might not be mutually exclusive.

There is one study available that analyzes within-ranks gender spillover effects on individual performance in a male-dominated setting: While Pazy and Oron (2001) find positive within-ranks effects for women’s performance, my study reveals insignificant cubic within-ranks effects for women’s performance. Yet, I find a significant cubic relationship for men’s performance with regards to a growing share of female peers. In contrast, Pazy and Oron (2001) show that men do not change their performance depending on gender group composition. While numerous studies on downward-flowing gender spillover effects reveal positive effects for women, e.g., when it comes to promotion (e.g., Matsa and Miller, 2011; Kunze and Miller, 2017) and pay (e.g., Cohen and Huffman, 2007; Cardoso and Winter-Ebmer, 2010), my study finds that the opposite is true for this particular industry sector. However, to the best of my knowledge there is no prior study analyzing both kinds of gender spillover effects on the individual performance outcome variable.

My findings have various implications for the efforts to get more women into traditionally male-dominated fields. Against the background that Norway was one of the first countries that introduced a female quota of 40% for board representation, my finding on within-ranks effects for men’s performance indicates that 40% might be a suitable quota bearing in mind that around this female peer share male performance is at a high level. Furthermore, my result on within-ranks effects for male performance generally confirms the suitability of a female quota since gender proportion is estimated to be linked to (men’s) individual performance and a critical mass of women is needed for a positive effect. Yet, my analysis reveals that a quota might not be enough: The negative downward-flowing effects contradict the results of former studies that largely found positive ones for other industry settings and outcome variables (e.g., Matsa and Miller, 2011; Devicienti et al., 2019). Instead, the majority of studies on downward-flowing spillover effects shows the phenomenon “women helping women.” Hence, the fact that I find negative downward-flowing effects might be due to the imbalanced gender ratios that are salient in this type of industry and need to be offset. The other studies’ results might imply that it may need a critical mass of female supervisors in an industry to overcome negative spillover effects. The female boss share in my data is 20.1% revealing that a female supervisor still is the exception in this industry. Yet, in order to change this, it is not only recommendable to rely on quota but to start even earlier, when it comes to attracting female students for an education in STEM-disciplines and offering special management courses for them. My findings might imply that quota need time to work out and are not only applicable at the executive level but should be implemented deep throughout the organization. While quota rely on the idea that women helping women, they may not work effectively in the industry setting that I analyzed with respect to spillover effects on individual performance. My results imply that the mere presence of female leaders does not improve men’s and women’s individual performance levels in a male-dominated industry during the research period. One reason might be that they are simply not enough.

From a business point of view, those companies and their personnel managers, in particular, that are in a male-dominated industry should be mindful of the fact that gender group composition might be linked to individual performance. As a result, my findings might have practical implications, for example, when personnel managers are confronted with the task of staffing a team. When it comes to the negative downward-flowing effects, Derks et al. (2011b) postulate that the queen bee syndrome emerges due to sexist organizational cultures with low gender identification. Strongly worded, just opening up the gates and inviting female leaders to a male-dominated industry might not be enough. Corporate leadership tutorials on gender-specific communication styles might help to gain understanding and break open an encrusted, masculine leadership culture that would ultimately rebound to an individual’s performance.

### Limitations and Future Research

In conclusion, some caution is warranted when it comes to generalizing my findings. For this study, I make assumptions that are not necessarily applicable to every background. First of all, I interpret individual performance as being purely actively driven by an individual. In reality, the often-unobserved side factors and biases of a supervisor who is responsible for evaluating a subordinate’s performance play a decisive role (e.g., Ibarra et al., 2010). As a matter of fact, having data on team performance and attrition rates would have complemented the study. Unfortunately, this piece of information is not available. Endogeneity issues also cannot entirely be ruled out because there might be relevant factors that I could not control for (omitted variable bias). Furthermore, the relationship between the growing share of female peers or female supervisors and the individual performance of men and women might not be depicted correctly by my study. The higher individual performance of women might also lead to a higher share of female peers or supervisors and vice versa (reverse causality). Also, information on the working hours of an individual worker is missing (part-time versus full-time). Nevertheless, other than these limitations, my analysis reveals novel insights into a topic that has been neglected by research thus far and is, therefore, of utter importance.

My study suggests that gender spillover effects are not only dependent on the outcome variable that is analyzed but also on the industry setting and its gender ratio. For future research, gender spillover effects studies on different industry settings with varying gender ratios would be useful to compare results and draw meaningful implications. Also, research differentiating between short-term and long-term effects would complement my findings in order to better understand the dynamics of spillover effects over time.

When it comes to the theoretical models on gender spillover effects, my results largely confirm the applied theory. Yet, this is not true for within-ranks effects on women’s performance. While my results suggest that this relationship can be rather described as being quadratic, the prevailing theory by Lazear (1999a) and Lazear (1999b) predicts a cubic link. Hence, within-ranks effects on individual performance do not follow the same rationale for men and women in a male-dominated sector and should be a subject for further theoretical considerations and extensions.

Ultimately, my work on gender spillover effects in this study can be regarded as a starting point for understanding this phenomenon. More work on the dynamics and mechanisms of these effects should follow.

## Data Availability

Requests to access the data sets should be directed to “charlotte.kraeft@uni-paderborn.de.”

## Footnotes

1 Standardized values for hierarchical ranks were used. Hence, they are comparable across companies.

2 See Supplementary Appendix A.1 for the correlation matrix of all relevant variables. As expected, age and rank as well as female peer and female boss share and rank show little higher degree of correlation.

3 See Supplementary Appendix A.2, A.3 for the results of the Hausman specification test.
